# Autoimmune Encephalitis - a diagnosis not to be overlooked in cases of psychosis

**DOI:** 10.1192/j.eurpsy.2025.718

**Published:** 2025-08-26

**Authors:** F. Ribeirinho Soares, A. Buinho, T. Oliveira, M. Fraga, J. Facucho

**Affiliations:** 1 Departamento de Saúde Mental, Hospital de Cascais, Cascais, Portugal

## Abstract

**Introduction:**

Autoimmune encephalitis (AE) is a neuroinflammatory condition that often presents with psychiatric symptoms, such as psychosis, mood disturbances, and cognitive impairment, mimicking psychiatric disorders like schizophrenia or bipolar disorder. These symptoms frequently appear before neurological signs, complicating diagnosis and delaying treatment. Early recognition of this condition is fundamental to improve patients outcomes.

**Objectives:**

This review examines the psychiatric manifestations of AE, highlights challenges in differentiating AE from primary psychiatric disorders, and emphasizes the importance of interdisciplinary collaboration.

**Methods:**

A nonsystematic review of literature focusing on the psychiatric presentation of AE.

**Results:**

AE often presents with psychiatric symptoms like hallucinations, delusions, agitation, and mood disturbances, frequently leading to misdiagnoses such as schizophrenia or bipolar disorder. In particular, anti-NMDA receptor encephalitis is associated with severe psychiatric manifestations like psychosis. Diagnosis is based on recognizing the rapid progression of psychiatric symptoms, along with seizures or other neurological signs, and confirming the autoimmune cause through specific autoantibody tests. Early treatment is essential to reverse these psychiatric symptoms. However, delayed diagnosis can result in persistent cognitive and psychiatric difficulties even after treatment. Diagnosing the psychiatric manifestations of AE is challenging due to their overlap with primary psychiatric disorders. However, the rapid onset and progression of symptoms, combined with neurological signs, should prompt clinicians to consider an autoimmune origin. Autoantibodies, particularly those against NMDA receptors, disrupt neurotransmitter systems, explaining the psychiatric features of AE. While psychotropic medications may provide temporary symptom relief, they do not target the underlying immune dysfunction. Timely immunotherapy significantly improves outcomes, and multidisciplinary collaboration between psychiatrists and neurologists is crucial for comprehensive patient care.

**Conclusions:**

AE should be considered in cases of acute psychosis, mood disturbances, or cognitive impairment. Early diagnosis and treatment are key to preventing long-term psychiatric and neurological issues. Greater awareness of AE’s psychiatric presentations aids in distinguishing it from primary psychiatric disorders and facilitates timely treatment. Further research is needed to explore the neuroimmune mechanisms behind AE and optimize treatment. Collaborative efforts between psychiatry and neurology are essential for successful patient outcomes.

**Disclosure of Interest:**

None Declared

